# Beyond Numbers: Decoding the Gendered Tapestry of Non-Communicable Diseases in India

**DOI:** 10.3390/ijerph21091224

**Published:** 2024-09-18

**Authors:** Farah Niazi, Abdul Rahique, Shyamkumar Sriram, Karuna Nidhi Kaur, Shazina Saeed

**Affiliations:** 1 Laboratory of Disease Dynamics & Molecular Epidemiology, Amity Institute of Public Health and Hospital Administration, Amity University, Noida 201303, India; fniazi.3107@gmail.com (F.N.); karunanidhisodhi@gmail.com (K.N.K.); 2 International Institute for Population Sciences, Mumbai 400088, India; rahique016@gmail.com; 3 Department of Rehabilitation and Health Services, College of Health and Public Service, University of North Texas, Denton, TX 76201, USA

**Keywords:** gender disparities, non-communicable diseases, public health, India, health policy

## Abstract

Introduction: Non-communicable diseases (NCDs) represent a major global health challenge, particularly in low- and middle-income countries like India, with significant gender disparities in mortality and disease burden. This study aims to investigate these disparities, using data from national health surveys, to inform gender-specific public health strategies and align with global health goals. Methodology: The study uses data from the Longitudinal Aging Study in India (LASI) and National Family Health Surveys (NFHS-4 and NFHS-5). Result: The results from the Longitudinal Ageing Study in India (LASI) and National Family Health Surveys (NFHS-4 and NFHS-5) indicate significant demographic and health-related variations among 65,562 participants. Key findings show gender disparities in lifestyle habits such as alcohol and tobacco use, and differences in health outcomes across age, education, and socioeconomic status. Notably, an increase in NCD prevalence, particularly hypertension and diabetes, was observed from NFHS-4 to NFHS-5, highlighting evolving health challenges in India. Conclusions: The study emphasizes the importance of gender in the prevalence and management of non-communicable diseases (NCDs) in India, advocating for public health strategies that address gender differences, socio-economic factors, and urban-rural disparities to achieve health equity.

## 1. Introduction

Non-communicable diseases (NCDs) are taking center stage in the rapidly changing global health landscape. These illnesses, which include diabetes, cancers, chronic respiratory diseases, and cardiovascular diseases, now represent a significant global health concern. According to the World Health Organization, NCDs account for a startling 74% of all deaths worldwide, pointing to a significant shift in health trends from infectious to chronic illnesses. Nonetheless, the effects of this change are not evenly experienced in all parts of the world. It is especially noticeable in low- and middle-income nations, such as India. The burden of noncommunicable diseases (NCDs) is disproportionately higher in these countries. This discrepancy can be attributed to a number of factors, including the fact that their healthcare systems are frequently inadequate for managing the chronic and complex nature of these diseases [1].

Significant changes in male/female differences in mortality rates and health outcomes have occurred over time and across different countries. In most countries, mortality rates have steadily decreased for both men and women for over a century, with male life expectancy consistently lower than female life expectancy [2]. Furthermore, the differences between male/female mortality rates are highly contingent on the circumstances in which people live and mortality-related epidemiological conditions, such as disease dominance, public health infrastructure, and healthcare resources [3]. Moreover, there are notable global differences in major cardiovascular risk factors between genders. Men are more prone to hypertension, whereas women generally exhibit higher lipid levels. Physiological markers also show higher inflammatory activity in women and a typically greater cardiovascular risk in men, though in certain contexts and times, women may have equal or higher risks. This underscores the need to investigate the relationship between non-communicable diseases (NCDs) and gender differences, to gather data that can guide the creation of gender-tailored recommendations in worldwide health strategies [4].

India, a country with a burgeoning population and a diverse socio-economic spectrum, exemplifies this challenge. The nation is currently experiencing a significant epidemiological transition characterized by a rising prevalence of NCDs, a trend that has been escalating in recent years. Factors contributing to this increase include urbanization, lifestyle changes, increased life expectancy, and associated risk factors such as tobacco use, unhealthy diets, physical inactivity, and harmful alcohol use. These shifts are evident in the growing incidence of conditions like hypertension, heart diseases, stroke, and diabetes [5]. The need for gender-related testing for NCD risk factors arises from the evidence suggesting variations in the prevalence of self-reported NCDs between men and women across the country. Gender, education, and income are associated with the prevalence of NCDs and their risk factors, leading to a catastrophic disease burden among vulnerable populations. Hence, understanding the gender-related differences in NCD risk factors and testing is crucial for designing effective public health interventions and policies that address the specific needs of different subgroups of the population [6].

Recent studies have shown that socio-economic factors such as income, education, and access to healthcare significantly influence the prevalence of NCDs, with lower socio-economic groups bearing a higher burden of disease. This socio-economic disparity is further exacerbated by gender differences, where women, particularly in rural areas, often have limited access to healthcare services and health education, leading to poorer health outcomes. Moreover, cultural norms and societal roles can influence health behaviors and access to healthcare, making it essential to consider these factors when designing public health interventions. Addressing these disparities requires a multifaceted approach that includes improving healthcare infrastructure, increasing health literacy, and implementing policies that address the unique needs of different demographic groups [7,8,9].

Another critical dimension to consider is the impact of urbanization and industrialization, which have significantly altered lifestyle patterns in India. The rapid growth of urban areas has led to changes in dietary habits, with increased consumption of processed and high-calorie foods. Sedentary lifestyles have become more common due to technological advancements and changes in occupational structures. These lifestyle modifications contribute to the rising incidence of NCDs. Furthermore, environmental factors such as air pollution, particularly in urban centers, have been linked to an increase in chronic respiratory diseases and cardiovascular conditions. The interplay between these environmental and lifestyle factors with socio-economic and gender disparities creates a complex web of determinants influencing the health outcomes related to NCDs in India [10,11,12,13,14].

The necessity of this study is underscored by the alarming trends and the multifaceted challenges associated with NCDs in India. While numerous studies have addressed the overall burden of NCDs, there is a paucity of research focusing on the gender-specific aspects of NCD prevalence and the socio-economic and cultural factors that influence these patterns. The gender-specific analysis of NCDs is essential to identify unique risk factors and health outcomes for men and women, which can differ significantly due to biological, social, and behavioral factors. Furthermore, with the goal of reducing health disparities and achieving the Sustainable Development Goals (SDGs), there is a pressing need for detailed, context-specific data that can inform targeted interventions [15,16].

This study therefore aimed to examine the intricate interplay between gender dynamics and NCDs in India. By exploring the association between NCDs and gender disparities, we aim to generate evidence that can inform the development of more gender-specific recommendations for global strategies, aligning with the target set by the Sustainable Development Goals (SDGs) to reduce NCD-related mortality. To achieve this, the study leverages data from the National Family Health Surveys (NFHS-4 and NFHS-5) and the Longitudinal Aging Study in India (LASI).

### Research Hypotheses

This study is guided by the following hypotheses:

**Hypothesis** **1.** *Gender disparities exist in the prevalence of non-communicable diseases (NCDs) in India.*

**Hypothesis** **2.** *Socioeconomic status significantly influences the prevalence of NCDs.*

**Hypothesis** **3.** *Urban-rural residence is associated with differences in the prevalence of NCDs.*

**Hypothesis** **4.** *Education level is inversely related to the prevalence of NCDs.*

## 2. Materials and Methods

### 2.1. Data Source and Sampling Design

The study utilized data from Wave 1 of the Longitudinal Ageing Study in India (LASI), conducted in 2017–2018. LASI represents a comprehensive national survey aimed at exploring the health, economic, and social dynamics associated with population ageing in India. A multistage stratified area probability cluster sampling design was employed to identify the study’s observational units. The primary focus was on adults aged 45 years and above, along with their spouses of any age.

Additionally, data from the National Family Health Survey (NFHS) rounds 4 and 5 were utilized to supplement and corroborate the findings from LASI.

### 2.2. Sampling Procedure

The sampling process for LASI differed between rural and urban areas. In rural regions, a three-stage design was implemented, while urban areas followed a four-stage design. The initial stage involved selecting Primary Sampling Units (PSUs), typically sub-districts. The subsequent stage comprised selecting villages in rural areas and wards in urban areas within the PSUs. For rural settings, households were chosen in the third stage directly from these villages. In contrast, urban areas introduced an additional stage where one Census Enumeration Block (CEB) was selected in each area, followed by household selection from the chosen CEB. The approach aimed to ensure a representative sample at every stage.

### 2.3. Model Specification and Description of Variables

The study employed logistic regression models to analyze the prevalence of non-communicable diseases (NCDs) such as hypertension and diabetes. The dependent variables (outcome variables) are the binary indicators of the presence of hypertension and diabetes among the participants. The independent variables (predictors) include demographic factors, socioeconomic status, place of residence, caste, religion, and lifestyle factors.

#### 2.3.1. Dependent Variables

Hypertension: variable indicating whether the participant has hypertension or not.

Diabetes: Binary variable indicating whether the participant has diabetes or not.

#### 2.3.2. Independent Variables

The independent variables in this study include several demographic and socioeconomic factors. Age is categorized into three groups: 45–59 years, 60–79 years, and 80+ years. Gender indicates male or female. Education levels range from ‘No Education’ to ‘Professional Degree/Course’. Socioeconomic status is measured by Monthly Per Capita Consumption Expenditure (MPCE) quintiles: Poorest, Poorer, Middle, Richer, and Richest [17]. Place of residence indicates rural or urban. Caste includes ‘Scheduled Caste’, ‘Scheduled Tribe’, ‘Other Backward Class’, and ‘Others’. Religion includes ‘Hindu’, ‘Muslim’, ‘Christian’, ‘Sikh’, and ‘Others’. Lifestyle factors are represented by alcohol consumption (Yes/No) and tobacco consumption (Yes/No).

### 2.4. Statistical Analysis

Descriptive statistics, including means and percentages, were calculated to summarize the data. Additionally, we used chi-square tests to assess the associations between categorical variables and logistic regression models to examine the relationships between independent variables (such as age, gender, education, socioeconomic status) and the dependent variables (hypertension and diabetes). *p*-value calculations were utilised to indicate the statistical significance of our findings. The *p*-value represents the probability that the observed results occurred by chance. In this study, we used a significance level of 0.05, meaning that a *p*-value below this threshold indicates a statistically significant result. All analyses were conducted using STATA version 15.1.

#### Logistic Regression Analysis

Logistic regression was employed to examine the relationship between demographic, socioeconomic, and lifestyle factors and the prevalence of hypertension and diabetes. This modeling approach was chosen because it is well-suited for binary outcome variables and can handle multiple predictor variables simultaneously. Logistic regression provides odds ratios, which are useful for interpreting the strength and direction of associations between predictors and outcomes. This method is advantageous in public health research for its ability to adjust for confounding variables and estimate the probability of the occurrence of an event.

## 3. Results

### 3.1. Longitudinal Ageing Study in India (LASI)

Table 1 presents an overview of the socio-economic and health profile of 65,562 study participants, encompassing 30,479 men and 35,083 women. The age distribution revealed a majority in the 45–59 age group (52.01%). Varied educational levels were observed, with a notable proportion having completed less than primary school (21.52%). Employment status indicated that 63.88% were currently working. Lifestyle habits showed 16.64% alcohol consumers and 34.6% tobacco users, with a higher prevalence among men in both cases. The religious composition was predominantly Hindu (73.32%), and caste distribution included 17.3% scheduled caste and 17.97% scheduled tribe participants. Additionally, 64.41% resided in rural areas, and economic status, measured by MPCE, spanned from the poorest (19.6%) to the richest (19.85%).

The study revealed varying prevalence rates of bone and joint diseases among older adults in different demographic categories. In the 80+ age group, the prevalence was 18.00%. Lower educational levels, not working or being retired, and tobacco consumption were associated with higher prevalence rates, ranging from 9.76% to 23.97%. Significant variations were observed across religious groups, with prevalence rates ranging from 8.62% to 17.22%. Caste, urban/rural residence, and economic status also exhibited notable differences, with prevalence rates ranging from 8.13% to 18.32%. Gender differences were evident in specific categories.

All reported *p*-values were less than 0.001, indicating strong statistical significance. A *p*-value less than 0.001 means that there is less than a 0.1% probability that the observed differences occurred by chance. This high level of statistical significance suggests that the associations between demographic factors and the prevalence of bone and joint diseases are highly unlikely to be due to random variation (Table 2).

### 3.2. National Family Health Survey-4 (NFHS-4)

**Figure 1:**
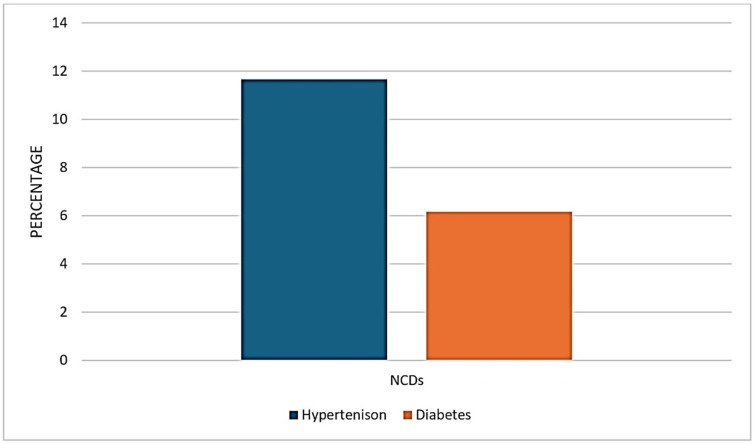
Prevalence of NCDs in NFHS-4 data.

The prevalence of non-communicable diseases (NCDs) in NFHS-4 for hypertension and diabetes among men was 15.89% and 8.17% respectively. Among women, the prevalence was 10.88% for hypertension and 5.75% for diabetes (Figure 1).

### 3.3. National Family Health Survey-5 (NFHS-5)

**Figure 2:**
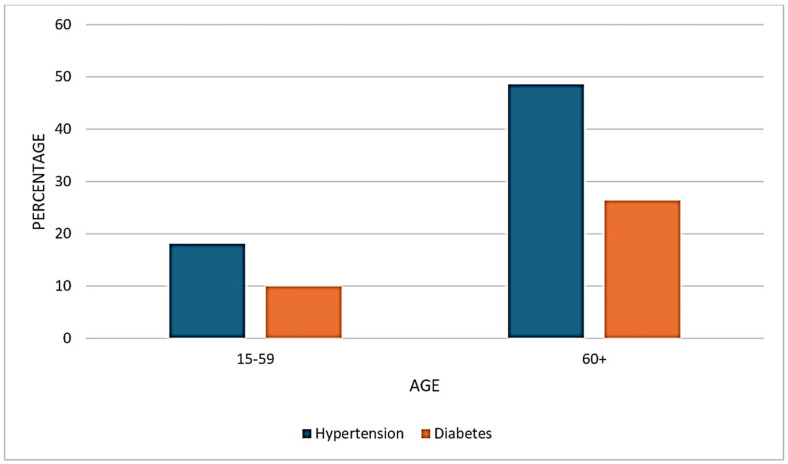
Prevalence of NCDs in NFHS-5 data.

The prevalence of non-communicable diseases (NCDs) in NFHS-5, categorized by age, indicated that among individuals aged 15–59, the prevalence was 18.08% for hypertension and 9.93% for diabetes. In the 60+ age group, the prevalence significantly increased to 48.56% for hypertension and 26.38% for diabetes (Figure 2).

**Figure 3:**
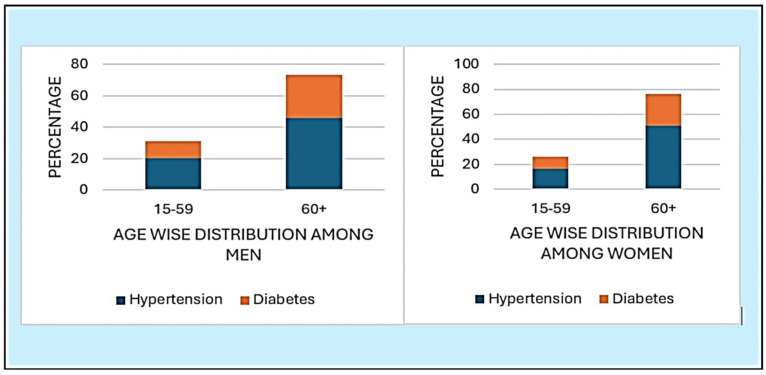
Prevalence of NCDs among Men and Women and the Age wise Distribution in NFHS-5 data.

For men aged 15–59, the prevalence of hypertension and diabetes was 20.26%, 10.81%, respectively. In the 60+ age group, the prevalence increased to 46.15% for hypertension and 27.19% for diabetes. Among women aged 15–59, the prevalence was 16.24% for hypertension and 9.19% for diabetes. In women aged 60+, the prevalence rose to 50.99% for hypertension and 25.55% for diabetes (Figure 3).

The logistic regression model for hypertension revealed lower odds for individuals with primary (OR: 0.8589), secondary (OR: 0.5358), and higher education (OR: 0.4222) compared to those with no education. Women exhibited lower odds (OR: 0.746) than men, and wealth index showed a positive association with increasing odds from the poorest to the richest category. Urban residence was associated with lower odds (OR: 0.8725) compared to rural areas. Caste, religion, and age also displayed significant associations. Similarly, the diabetes model indicated lower odds for individuals with primary (OR: 0.9748), secondary (OR: 0.6573), and higher education (OR: 0.5044) compared to those with no education. Women had lower odds (OR: 0.777) than men, and wealth index exhibited a positive association with increasing odds from the poorest to the richest category. Urban residence was associated with lower odds (OR: 0.8514) compared to rural areas. Caste, religion, and age demonstrated significant associations in both models (Table 3 and Table 4).

**Figure 4:**
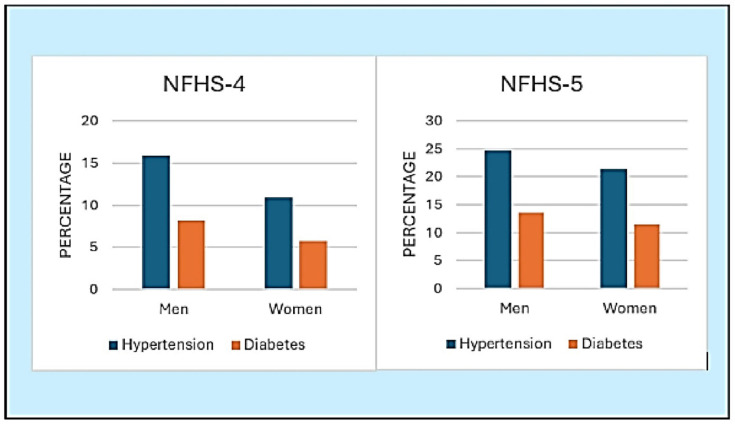
A comparison of prevalence of NCDs among men and women in NFHS-4 and NFHS-5 data.

On comparison of findings from NFHS-4 and NFHS-5, an increase in the prevalence of hypertension and diabetes is observed among both men and women in NFHS-5. Specifically, in NFHS-5, the prevalence of hypertension was 24.69% for men and 21.34% for women, compared to 15.89% and 10.88% in NFHS-4, respectively. Similarly, the prevalence of diabetes increased in NFHS-5, with 13.52% for men and 11.49% for women, compared to 8.17% and 5.75% in NFHS-4 (Figure 4).

### 3.4. Gender Disparities in Lifestyle Habits and Health Outcomes

The study revealed significant gender disparities in lifestyle habits and health outcomes among the participants. Men were more likely to consume alcohol (34.09%) and use tobacco (55.92%) compared to women (4.12% and 20.03%, respectively), with logistic regression confirming lower odds for women in both behaviors. Health outcomes also varied by gender: hypertension prevalence was higher in men in NFHS-4, but NFHS-5 showed increased rates among women, with 16.24% of women aged 15–59 and 50.99% of women aged 60+ having hypertension, compared to 20.26% and 46.15% of men, respectively. Diabetes followed a similar pattern, with men having slightly higher prevalence rates, though the gap narrowed among the older population. Higher education levels were associated with lower odds of hypertension and diabetes, particularly among women, and urban residents, especially women, showed lower odds of hypertension compared to rural residents. These findings highlight the need for gender-sensitive public health strategies to address the unique health risks and behaviors of men and women.

## 4. Discussion

Our study provided a comprehensive analysis of the socio-economic and health profiles of 65,562 participants, with a particular focus on gender disparities in the prevalence of non-communicable diseases (NCDs) in India. The data from NFHS-4, NFHS-5, and the Longitudinal Ageing Study in India (LASI) offered critical insights into how these disparities manifest across different demographic segments.

The study’s findings revealed notable gender disparities in the prevalence of NCDs in India. Initially, NFHS-4 data showed higher rates of hypertension and diabetes among men, likely linked to traditional lifestyle factors such as higher tobacco and alcohol use, and occupational stress. However, a significant shift was observed in NFHS-5, with an alarming increase in these conditions among both genders, particularly among women [18].

This rise in NCD prevalence among women suggests changes in lifestyle patterns, socio-economic roles, and increased stress levels. The evolving role of women in the workforce and the dual burden of professional and domestic responsibilities may contribute to this trend. Additionally, changes in dietary habits and increased sedentary lifestyles are potential risk factors [19].

These findings underscore the need for gender-specific public health strategies. Tailored interventions should address the unique lifestyle, stress, and health awareness factors affecting both men and women. This shift highlights the importance of re-evaluating public health policies to effectively manage the evolving landscape of NCDs in India, with a particular focus on the changing health needs of women.

The data also hint at a socio-economic transition in India, where health challenges are shifting from infectious diseases to NCDs, impacting men and women differently due to their distinct socio-economic experiences and empowerment levels [20].

This study revealed that age is a significant factor in the prevalence of bone and joint diseases among older adults. The data indicates that the prevalence rates increase with age. This trend underscores the heightened vulnerability of older adults to bone and joint conditions as they age. The higher prevalence of bone and joint diseases among older adults, especially in the 80+ age group, was linked to socio-demographic factors such as lower educational levels and tobacco consumption. These findings indicate that gender disparities in health are further compounded by socio-economic status and lifestyle choices, highlighting the need for gender-sensitive health interventions [21].

The logistic regression models from NFHS-5 revealed a significant association between higher educational levels and reduced odds of hypertension and diabetes. This protective effect of education was particularly evident among women, highlighting the potential of educational empowerment as a crucial strategy in addressing gender disparities in health. Education likely contributes to better health literacy, leading to healthier lifestyle choices and improved management of health conditions [22].

Furthermore, the positive correlation between the wealth index and NCDs suggests that lifestyle-related risk factors, such as sedentary behavior and unhealthy diets, are more prevalent in higher economic strata. This trend affects both genders but has different implications for each. For instance, higher-income men might be more prone to stress-related conditions due to work pressures, while women in the same economic bracket might face lifestyle challenges related to diet and physical inactivity [23].

These findings underscore the need for targeted health interventions that consider both educational and economic backgrounds. For women, particularly, enhancing access to education could be a pivotal step in reducing health disparities. For higher-income groups, interventions might focus more on addressing lifestyle-related risk factors to mitigate the rising prevalence of NCDs.

The study’s findings indicated a notable urban-rural divide in health outcomes, with urban residents showing lower odds of hypertension compared to their rural counterparts. This disparity was evident in both genders but was more pronounced among women. The lower incidence of hypertension in urban areas could be attributed to better healthcare access, greater health awareness, and possibly more health-promoting lifestyle choices prevalent in urban settings [24].

The pronounced disparity among rural women highlights the compounded challenges they face, including limited access to healthcare services, lower health literacy, and potentially higher levels of physical labor. These factors contribute to the increased vulnerability of rural women to hypertension and other health issues [25].

This urban-rural health divide underscores the need for targeted health policies focused on rural areas, particularly for women. Interventions in these regions should not only aim to improve healthcare access but also focus on health education and the promotion of healthier lifestyle practices. Addressing these disparities is crucial for achieving equitable health outcomes across different demographics in India [26].

Previous studies have documented the prevalence of NCDs across various demographics. However, this research uniquely highlights a concerning rising trend of NCDs among women in India, indicating a shift in gender dynamics and health burdens [5,6]. This observation contrasts with another study, which reported a higher cardiovascular risk among men globally [4]. The data from NFHS-5 reveals an alarming increase in hypertension and diabetes among women, highlighting the urgent need for targeted public health interventions. The observed gender disparities in health outcomes are also in line with findings from the Global Burden of Disease Study, which underscores the importance of considering gender in health policy formulation [27,28].

Several other studies have pointed out that the prevalence of hypertension and diabetes is generally higher in urban areas due to lifestyle factors such as higher stress levels, sedentary behavior, and dietary habits [29,30,31]. However, our study indicates that while urban women show lower prevalence rates for these conditions compared to their rural counterparts, there is still a significant rise in NCDs among urban women, suggesting that urbanization alone does not protect against these diseases. This finding is consistent with research from other low- and middle-income countries where urbanization has been linked to both positive and negative health outcomes [32,33].

Additionally, the findings on the protective effect of education against NCDs align with another study, which emphasized the importance of health literacy in managing chronic diseases [24]. Studies in China and Brazil have demonstrated similar patterns where education and income levels significantly impact NCD prevalence and management [8,34].

This convergence of evidence highlights the multifaceted nature of NCDs and the necessity for comprehensive strategies to address gender-specific health challenges.

### 4.1. Implications for Gender-Sensitive Public Health Policy

The study’s findings highlight a critical need for public health policies that specifically tackle gender disparities in non-communicable diseases (NCDs). Key areas of focus should include:

#### 4.1.1. Gender-Tailored Awareness Programs

Develop awareness initiatives that are specifically designed for men and women, considering the unique risk factors, symptoms, and challenges each gender faces in relation to NCDs. This approach ensures that health messages are more relevant and effective.

#### 4.1.2. Enhanced Healthcare Access in Rural Areas

Prioritize improving healthcare accessibility in rural regions, with a special emphasis on women. This effort could involve increasing the number of healthcare facilities, providing mobile health services, and ensuring the availability of female healthcare professionals to address gender-specific health needs.

#### 4.1.3. Gender-Sensitive Health Education and Prevention

Implement health education and preventive measures that are sensitive to gender differences. This could include programs on healthy lifestyle choices, stress management, and regular health screenings, all tailored to address the distinct health concerns and societal roles of men and women.

By focusing on these areas, public health policies can more effectively reduce gender disparities in NCDs and promote overall health equity.

#### 4.1.4. Limitations and Future Research Directions

This study’s strengths include its comprehensive analysis of gender disparities in the prevalence and management of non-communicable diseases (NCDs) in India, utilizing robust data from the Longitudinal Aging Study in India (LASI) and the National Family Health Surveys (NFHS-4 and NFHS-5). The large sample size and inclusion of multiple demographic variables enhance the generalizability of our findings and provide valuable insights into the socio-economic and health profiles of a diverse population. Additionally, the study’s focus on gender-specific public health strategies addresses a critical gap in the existing literature.

However, several limitations should be acknowledged. The reliance on self-reported data may introduce reporting biases and affect the accuracy of the findings. Moreover, the cross-sectional design of the surveys limits our ability to establish causal relationships between the observed factors and NCD prevalence. The study also did not account for potential confounding variables such as genetic predisposition and access to healthcare services, which could influence the results.

Future research should aim to address these limitations by incorporating longitudinal data to examine the temporal relationships between risk factors and NCD outcomes. Additionally, exploring the impact of cultural and societal norms on gender-specific health behaviors could provide deeper insights into addressing these disparities. Further studies should also investigate the effectiveness of targeted public health interventions and educational programs in reducing NCD prevalence among different demographic groups. By addressing these areas, future research can contribute to developing more effective and equitable health strategies for managing NCDs in India.

## 5. Conclusions

The study underscores the critical role of gender in the prevalence and management of non-communicable diseases (NCDs) in India. The significant rise in NCD prevalence among women, as revealed by NFHS-5 data, indicates a shift that demands gender-specific public health strategies. Notable gender disparities were observed in lifestyle habits and health outcomes, with men exhibiting higher tobacco and alcohol use, but women showing a rising trend in hypertension and diabetes. This shift is particularly pronounced in rural areas where women face compounded challenges due to limited healthcare access and lower health literacy.

The findings highlight the urgent need for gender-sensitive health policies and interventions. Public health strategies should focus on enhancing healthcare access in rural areas, promoting health education, and addressing the unique health risks faced by women. The protective effect of education observed in the study underscores the importance of educational empowerment as a crucial strategy in mitigating NCD risks.

The study calls for a nuanced approach to understanding and addressing the complex interplay of gender roles, socio-economic status, education, and urban-rural divides in health outcomes. Effective public health strategies must recognize and address these gender disparities to ensure equitable health for all. Addressing these issues through targeted interventions, improved healthcare access, and enhanced educational initiatives will be crucial in mitigating the rising NCD burden and achieving health equity in India.

## Figures and Tables

**Table 1:** Socio-economic and health profile of study participants.

Background Variable	Total (N = 65,562)		Men (N = 30,479)		Women (N = 35,083)	
	N	%	N	%	N	%
Age						
45–59	34,098	52.01	15,381	50.46	18,717	53.35
60–79	28,075	42.82	13,506	44.31	14,569	41.53
80+	3389	5.17	1592	5.22	1797	5.12
Education						
Less than Primary School	7478	21.52	4059	19.33	3419	24.87
Primary School completed	8618	24.81	4722	22.49	3896	28.34
Middle School completed	6241	17.96	3853	18.35	2388	17.37
Secondary School completed	5885	16.94	3830	18.24	2055	14.95
Higher Secondary/Intermediate	2803	8.07	1917	9.13	886	6.45
Diploma	323	0.93	244	1.16	79	0.57
Graduate Degree	2218	6.38	1538	7.33	680	4.95
Postgraduate	679	1.95	470	2.24	209	1.52
Professional Degree/Course	496	1.43	362	1.72	134	0.97
Working Status						
Working	32,541	63.88	19,386	66.35	10,344	56.4
Not Working	15,372	30.18	7269	24.88	7541	41.11
Retired	3028	5.94	2563	8.77	457	2.49
Alcohol Consumption						
Yes	11,922	16.64	10,282	34.09	1436	4.12
No	59,709	83.36	19,883	65.91	33,407	95.88
Tobacco Consumption						
Yes	24,777	34.6	16,864	55.92	6978	20.03
No	46,836	65.4	13,292	44.08	27,854	79.97
Religion						
Hindu	52,973	73.32	22,450	73.66	25,649	73.12
Muslim	8667	12	3549	11.64	4254	12.13
Christian	7215	9.99	3002	9.85	3534	10.07
Sikh	1999	2.77	878	2.88	988	2.82
Buddhist	529	0.73	222	0.73	257	0.73
Jain	176	0.24	77	0.25	74	0.21
Jewish	5	0.01	2	0.01	3	0.01
Parsi/Zoroastrian	7	0.01	4	0.01	3	0.01
Others	529	0.73	229	0.75	251	0.72
None	145	0.2	66	0.22	66	0.19
Caste						
Scheduled caste	12,046	17.3	5049	17.16	5910	17.48
Scheduled tribe	12,509	17.97	5256	17.86	6109	18.07
Other backward class (OBC)	27,184	39.04	11,521	39.16	13,108	38.77
Others	17,887	25.69	7597	25.82	8682	25.68
Place of Residence						
Rural	64.41	64.41	19,889	65.25	22,535	64.23
Urban	25,716	35.59	10,590	34.75	12,548	35.77
MPCE						
Poorest	14,158	19.6	5965	19.57	6976	19.88
Poorer	14,530	20.11	6086	19.97	7104	20.25
Middle	14,537	20.12	6105	20.03	7058	20.12
Richer	14,686	20.33	6206	20.36	7004	19.96
Richest	14,339	19.85	6117	20.07	6941	19.78

**Table 2:** Prevalence Estimates (%) of Bone and Joint Diseases among Older Adults.

Age	Total (%)	Men (%)	Women (%)	*p*-Value
45–59	11.81	7.96	14.97	**<0.001**
60–79	17.77	14.53	20.76	**<0.001**
80+	18.00	16.49	19.34	**<0.001**
Education				
Less than Primary School	15.57	13.23	18	**<0.001**
Primary School completed	14.19	12	16	**<0.001**
Middle School completed	12.34	9.53	16	**<0.001**
Secondary School completed	13.27	10.37	17.13	**<0.001**
Higher Secondary/Intermediate	11.06	8.09	15	**<0.001**
Diploma	11.47	7.82	20.62	**<0.001**
Graduate Degree	11.39	8.48	16.17	**<0.001**
Postgraduate	9.76	9.15	10.79	**<0.001**
Professional Degree/Course	12.38	9.97	17.44	**<0.001**
Working Status				
Working	10.32	9.1	12.13	**<0.001**
Not Working	19.04	16.38	21.43	**<0.001**
Retired	15.1	13.5	23.97	**<0.001**
Alcohol Consumption				
Yes	11.41	10.97	14.21	**<0.001**
No	14.66	11.45	16.26	**<0.001**
Tobacco Consumption				
Yes	13	11.34	15.34	**<0.001**
No	15	11.25	16.37	**<0.001**
Religion				
Hindu	14.51	11.72	17	**<0.001**
Muslim	17.22	12	19.51	**<0.001**
Christian	8.62	13.91	10.13	**<0.001**
Sikh	12.95	6.53	15.98	**<0.001**
Buddhist	10.63	9.11	12.54	**<0.001**
Jain	13.07	8.04	17.17	**<0.001**
Jewish	0	7.79	0	**<0.001**
Parsi/Zoroastrian	14.29	0	12.54	**<0.001**
Others	6.06	0	17.17	**<0.001**
None	10	7.69	11	**<0.001**
Caste				
Scheduled caste	14	11	15.8	**<0.001**
Scheduled tribe	8.13	6.97	8.98	**<0.001**
Other backward class (OBC)	15.82	13.23	17.74	**<0.001**
Others	15.28	11.16	18.32	**<0.001**
Place of Residence				
Rural	13.56	11.65	15	**<0.001**
Urban	15.08	10.61	18.22	**<0.001**
MPCE				
Poorest	11.63	10	13	**<0.001**
Poorer	13.1	10.86	15	**<0.001**
Middle	13.92	12.74	16	**<0.001**
Richer	15.4	12.85	17	**<0.001**
Richest	16.41	11.29	19	**<0.001**

**Table 3:** Logistic Regression Model on Hypertension.

Covariates	OR	*p* > |z|	[95% Conf. Interval]	
Education				
No Education	1		0.8487579	0.869198
Primary	0.8589171	0.000	0.5303529	0.541234
Secondary	0.5357658	0.000	0.415857	0.428709
Higher	0.4222342	0.000		
Gender				
Men	1			
Women	0.7459539	0.000	0.7401386	0.751815
Wealth Index				
Poorest	1			
Poorer	1.182756	0.000	1.168399	1.197289
Middle	1.413119	0.000	1.395466	1.430994
Richer	1.669806	0.000	1.64764	1.692269
Richest	2.029879	0.000	1.999445	2.060777
Area				
Urban	1			
Rural	0.8725476	0.000	0.8640066	0.881173
Caste				
SC	1			
ST	1.123592	0.000	1.108293	1.139101
OBC’S	1.053591	0.000	1.042373	1.06493
Others	1.175259	0.000	1.160964	1.18973
Religion				
Hindu	1			
Muslim	0.8486175	0.000	0.8372453	0.860144
Christian	1.223402	0.000	1.203909	1.243211
Sikh	1.411758	0.000	1.380021	1.444224
Others	1.408715	0.000	1.366481	1.452255
Age				
15–59	1			
60+	3.320056	0.000	3.289156	3.351247

**Table 4:** Logistic Regression Model on Diabetes.

Covariates	OR	*p* > |z|	[95% Conf. Interval]	
Education				
No Education	1			
Primary	0.9748	0.001	0.960675	0.989263
Secondary	0.6573	0.000	0.649033	0.665817
Higher	0.5044	0.000	0.495822	0.515257
Gender				
Men	1			
Women	0.777	0.000	0.769649	0.784808
Wealth Index				
Poorest	1			
Poorer	1.1352	0.000	1.117662	1.153174
Middle	1.3265	0.000	1.305642	1.347866
Richer	1.587	0.000	1.560768	1.613716
Richest	1.8611	0.000	1.826608	1.896319
Area				
Urban	1			
Rural	0.8514	0.000	0.841211	0.861739
Caste				
SC	1			
ST	0.9272	0.000	0.91085	0.943751
OBC’S	1.0969	0.000	1.082473	1.111654
Others	1.1295	0.000	1.112544	1.146895
Religion				
Hindu	1			
Muslim	0.8249	0.000	0.811234	0.838912
Christian	1.2041	0.000	1.179685	1.229079
Sikh	0.8866	0.000	0.860048	0.914144
Others	1.0518	0.019	1.008494	1.097093
Age				
15–59	1			
60+	2.6783	0.000	2.648211	2.708733

## Data Availability

The original contributions presented in the study are included in the article, further inquiries can be directed to the corresponding author.
